# Prevalence of Hookah Smoking among University Students in Iran: A Meta-Analysis of Observational Studies

**Published:** 2020-01

**Authors:** Mahmoud KHODADOST, Khadije MAAJANI, Abbas ABBASI-GHAHRAMANLOO, Morteza NASERBAKHT, Ebrahim GHODUSI, Fatemeh SARVI, Azar MOHAMMADZADEH, Seyed Abbas MOTEVALIAN, Ahmad HAJEBI

**Affiliations:** 1 Department of Epidemiology, School of Public Health, Iran University of Medical Sciences, Tehran, Iran; 2 Department of Epidemiology and Biostatistics, School of Public Health, Tehran University of Medical Sciences, Tehran, Iran; 3 Department of Public Health, School of Health, Ardabil University of Medical Sciences, Ardabil, Iran; 4 Mental Health Research Center, Iran University of Medical Sciences, Tehran, Iran; 5 Mental and Social Health Office, Ministry of Health, Tehran, Iran; 6 Department of Epidemiology, School of Health, Larestan University of Medical Sciences, Larestan, Iran; 7 Clinical Research Development Center, Aliasghar Hospital, Iran University of Medical Sciences, Tehran, Iran; 8 Research Center for Addiction & Risky Behaviors (ReCARB), Psychiatric Department, Iran University of Medical Sciences, Tehran, Iran

**Keywords:** Prevalence, Hookah, College students, Iran, Meta-analysis

## Abstract

**Background:**

The rise in popularity of waterpipe smoking among younger people cause increase its deleterious effects on health in recent years. The aim of this study was to estimate the pooled prevalence of water-pipe smoking in university students in Iran.

**Methods:**

We performed the literature search from 1946 to January 21, 2019, in several international and national databases such as Medline/PubMed, Web of Science, Scopus, Google Scholar, Magiran, Iranmedex, and IranPsych. To investigate the between-study heterogeneity we used the chi-squared test and I^2^ index. We used a random-effects model to estimate the pooled prevalence of water-pipe smoking. The potential source of heterogeneity was assessed by subgroup analysis and meta-regression.

**Results:**

According to the eligibility criteria, we included 37 relevant studies in our meta-analysis. The pooled prevalence of lifetime water-pipe smoking was 25% (95% CI: 22–29) and in male and female subgroups was 37% (95%CI: 30–45), 17% (95%CI: 15–19) respectively. The pooled prevalence of water-pipe smoking in last year was 21% (95%CI: 16–25) and in last month was 8% (95%CI: 5–11). Results of meta-regression analysis showed that there was not any significant association between suspected variables and the prevalence of water-pipe smoking.

**Conclusion:**

The higher prevalence rate of water pipe smoking among university students indicates the emergency need for planning preventive program.

## Introduction

Tobacco smoking is one of the preventable causes of morbidity and mortality in the world that contributes to over 5 million deaths every year (1). Tobacco-related deaths will rise from 6.4 million in 2015 to 8.3 million in 2030 (2). In different countries, tobacco is used in different forms.

In the Eastern Mediterranean Region (EMR) and the Far East, one of the traditional methods of tobacco using is hookah smoking. In this form, smoke is inhaled through a water reservoir. The hookah is also known as local names such as shisha, hubble-bubble, kalian, narghile, waterpipe, gona and arghilr (3).

Hookah smoking increases the risk of a variety of adverse health outcomes such as esophageal cancer, chromosomal aberrations, decreased pulmonary and cardiovascular function, low birth weight, infertility, dental problems and infectious diseases (4, 5). Among adults, the prevalence of hookah smoking was highest in the EMR, and this index for youth was highest between EMR and Europe regions (6). Among EMR countries, hookah smoking had a decreasing trend for the most part of the 20^th^ century. But in the starting 1990s, hookah smoking reemerged as a popular habit among youth at first in the EMR and then all over the world (7).

In different countries and studies, the prevalence of hookah smoking has been reported separately. For example, the prevalence of hookah smoking among university students reported as 21.1% in Lebanon(8), 8% in Saudi Arabia (9) (ever use among male medical students), and 54% ever and 33% current in Pakistan (10), 41% ever and 10% last month in USA (11), 38% ever and 8% regular use in UK (12).

In Iran, based on the results of two national health and disease evaluations in 1991 and 1999, the prevalence of hookah smoking had an increasing trend among 15–24 years old males and females (13). The prevalence of hookah smoking was 4.3% among 15–24 yr old participants in Iran (14). Hookah smoking is particularly popular among Iranian young adults and university students (3).

There are several factors that contribute to this popularity. First, emergence of flavored and sweetened tobacco has added to hookah’s popularity (15). Second, hookah smoking is relatively inexpensive. Third, there is a common misconception about hookah’s safety and most of students think that it is harmless (3). Fourth, there are a lot of accessible hookah bars and hookah cafes in Iranian cities and fifth, there are no legal prohibitions for hookah smoking in Iran.

Because of different prevalence rates of hookah smoking among Iranian university students we aimed to estimate the pooled prevalence of hookah smoking among this stratum of population by meta-analysis.

## Methods

### Study protocol

In this systematic review and meta-analysis, we identified studies that reported the prevalence of hookah smoking among university students in Iran. The preferred item for reporting of systematic review and meta-analysis (PRISMA) guidelines was used to design, conduct and report the result of study.

### Search strategy

We performed the literature search from 1946 to January 21, 2019 on several international online databases (Medline/PubMed, EMBASE, Web of Science, CINAHL, PsycINFO), and national (Magiran, Scientific Information Database, Iranmedex, Medlib, Irandoc, and IranPsych) databases. We used the PICO of systematic review and meta-analysis for retrieved and screen the related studies. Multiple combinations of keywords and phrases were used to specify the geographic location (i.e., country and province names), the population of interest (e.g., university students), medical domains (e.g., substance-related disorders), and type of substance (e.g., “Hubble-bubble” OR “Hookah” OR “wastepipe”). The following medical subject headings (MeSH) and non-MeSH keywords were used in our search strategy: “Hubble-bubble” OR “Hookah” OR “waterpipe” AND “Student” OR “university student” AND “Iran” AND “Prevalence” AND “Substance-Related Disorders” OR “Drug Abuse” OR “Drug Dependence” OR “Drug Addiction” OR “Substance Use Disorders”. We did not apply limitations on time of publication and language. We assess the reference section of relevant review studies or national program reports to identify studies and unpublished studies were excluded in the search strategy. EndNote X7 citation manager software (version X7, for Windows, Thomson Reuters, and Philadelphia, PA, USA) was used to manage and screen citations from several online databases.

### Eligibility criteria

We used the following criteria to select the eligible publications:

Inclusion criteria: 1) We included all observational studies reporting data on the prevalence of hookah smoking among University Students, including cross-sectional studies, cohort studies and case-control studies. 2) For the disease area, we included studies that report data on hookah smoking through self-rated questionnaires or interviews through interviews among university students. 3) We limited our geographic scope to studies conducted within Iran. 4) For the study population, we included studies conducted among university students at the time of the study.

Exclusion criteria: 1) We excluded reviews, meta-analyses, case series, case reports, short communication, comments, letters, ecologic and qualitative studies but retained them for hand searching of references. 2) Studies with a sample size less than 100 were considered underpowered and prone to a wider range of biases and thus were excluded. 3) Any studies that reported the prevalence of substance use among Iranian students residing aboard. 4) We excluded studies in the general population’s high school students and other age and gender-specific groups that not include university students.

### Data extraction

Two of the co-authors (AM & KM) independently extracted data from included studies using structured sheets in Microsoft Excel® and discussed disagreements with the third coauthor (MK) as indicated. We extracted data on 1) authors, 2) publication year, 3) publication type, 4) site/s of study, 5) study implementation year, 6) type of study, 7) sampling method, 8) study population (eligibility criteria) and sample size, 9) data gathering method (anonymous self-rated questionnaire, anonymous self-rated computer-based questionnaire, anonymous interview, data extraction from university students health files), 10) language of the publication (Farsi, English), 11) study scale (city, province, sub-national, national), 12) number of recruitment sites, 13) gender distribution, 14) age characteristics, 15) other key socioeconomic indicators, and 16) prevalence of water-pipe use.

### Quality assessment

After including the relevant studies according to inclusion criteria, we used strengthening the reporting of observational studies in epidemiology (STROBE) checklist (16) to investigate the quality of each eligible study. The studies were classified into three groups based on this checklist. If the studies get more than 80 percent of total score to consider as high quality, 60–79% of total score as intermediate quality and 30–59% of total score they classified as low quality. Two authors (KH, M, and M.KH) were independently investigating the quality of each included study. The agreements between reviewers were assessed by weighted Kappa (78%).

### Statistical analysis

We used the Q test and I^2^ index to investigate the statistical heterogeneity. Based on results of heterogeneity tests, we used random effect model weighted by the inverse of variance to calculate pooled estimates and 95% confidence intervals (CI) for prevalence of hookah smoking use. The standard error in each study was calculated using the binomial distribution. The metaprop package in Stata was used to calculate CIs for the original data using the exact binomial and score test. Meta-regression analysis was used to investigate the source of heterogeneity. The sensitivity analysis was performed using metainf command in stata software. Publication bias was not assessed, because the prevalence rate as a proportion always is a positive number and if we saw asymmetry in funnel plot it is not due to the publication bias. We used Stata11 (StataCorp, College Station, TX, USA) to perform all statistical analysis.

## Result

### Study characteristics

**Fig. 1:**
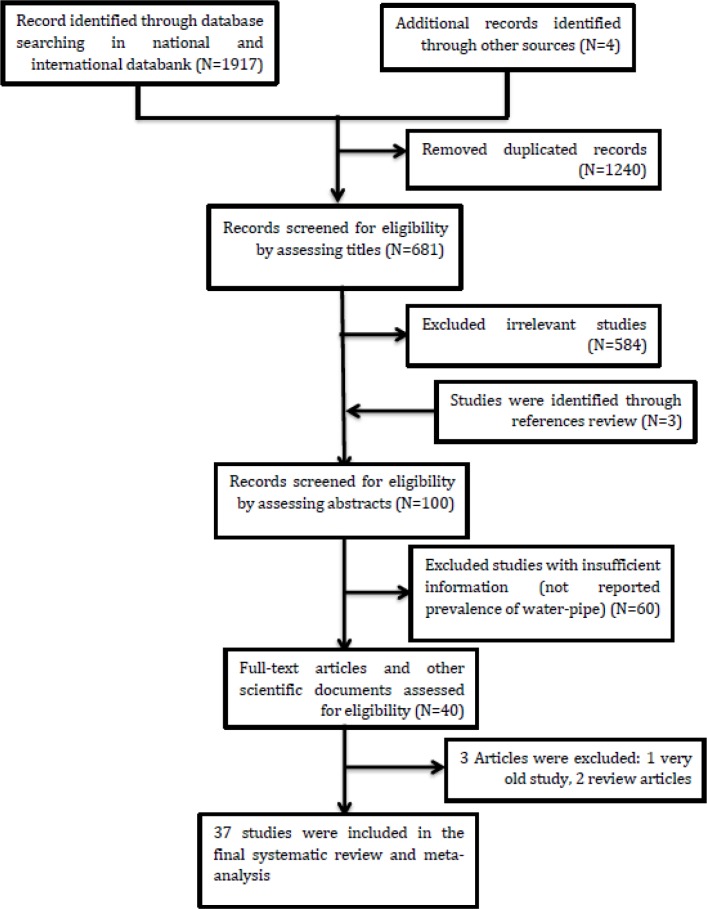
Flow diagram showing the different phases involved in searching for relevant publications in prevalence of Water-pipe smoking in college student in Iran

The result of the initial search yielded 1917 studies and after screening the included studies by title, abstract and full text according to the inclusion and exclusion criteria, 37 studies were finalized (Fig. 1). The characteristics of included studies are reported in Table 1.

**Table 1:** The characteristic of studies were included in the meta-analysis of water-pipe smoking among university students in Iran

	***Manuscript Reference number***	***Year of study***	***Study location***	***Age in years (Mean)***	***Sampling methods***	***Sample size (n)***	***Response rate (%)***	***Prevalence (%)***		
								**Last month**	**Last year**	**Lifetime**
1	(44)	2013	Tehran	21.1 (3.1)	Stratified random sampling	1992	-	8.9	17.8	26.6
2	(45)	2011	Iran	22	Random Cluster Sampling	8352	-	-	-	28.42
3	(46)	2011	Tabriz	22.1	Random Cluster Sampling	1838	-	-	-	8.5
4	(47)	2013	Tehran	23	Random sampling	604	-	-	-	29.3
5	(48)	2009	Yazd	22 (3.4)	Random sampling	534	-	-	-	15.9
6	(49)	2010	Zanjan	21.3 (2.3)	Stratified random sampling	1200	-	13	-	18.5
7	(50)	2013	Jahrom	21.2 (2.6)	Random sampling	1149	-	5.1	-	24.02
8	(51)	2015	Fasa	23.1 (2.5)	Stratified random sampling	157	-	-	-	32.3
9	(52)	2017	Tehran	21.3 (2.7)	Census	1012	-	-	-	34.1
10	(53)	2016	Larestan	22.3 (2.4)	Random sampling	390	100%	-	-	22.6
11	(54)	2017	Asadabad	22.7 (3.3)	Stratified random sampling	400	100%	-	-	32
12	(55)	2010	Bandar Abbas	23	Stratified random sampling	310	100%	-	-	24.8
13	(56)	2011	Tabriz	22.1 (2.2)	Random sampling	1837	100%	8.5	-	8.5
14	(17)	2013	Iran	22.5	Stratified random sampling	1053	100%	-	-	41.3
15	(57)	2014	Tehran	22.4	Random sampling	422	100%	-	-	14.9
16	(58)	2009	Tehran	20.2 (1.8)	Census	1568	100%	8.9	-	30.8
17	(21)	2016	kurdistan	_	Stratified random sampling	288	100%	-	-	11
18	(59)	2012	Zahedan	_	Random Cluster Sampling	1014	98.9%	-	-	40.4
19	(60)	2011	Tehran	22.9	Random Cluster Sampling	977	100%	-	-	27.7
20	(61)	2007	Isfahan & Kashan	_	Random sampling	812	100%	-	-	19.2
21	(18)	2008	Shiraz	_	Random sampling	971	100%	3.6	-	6.3
22	(62)	2006	Iran	22	Random Cluster Sampling	8373	99.9%	13	21	30
23	(63)	2011	Tehran	_	Random Cluster Sampling	3582	98.6%	-	-	25.7
24	(64)	2006	Tehran	_	Random sampling	2997	99.7%	13.2	22.1	33.9
25	(20)	2015	Lorestan	19.6 (2.2)	Random sampling	1131	95.8%	0.9	-	14
26	(65)	2008	Broujerd	23	Census	100	100%	-	-	36
27	(66)	2011	Iran	_	Stratified random sampling	7330	95.11%	11.6	17.9	28.7
28	(67)	2010	Kerman	_	Stratified random sampling	180	100%	-		38.3
29	(68)	2018	Iran	20.6 (2.4)	Random Cluster Sampling	4940	100%	-		17
30	(69)	2017	Qazvin	19.6 (2.4)	Census	524	97.9%	-		35.5
31	(70)	2018	Hormozgan	23 (4.2)	Multi stage random sampling	524	100%		14.5	_
32	(41)	2015	Tabriz	_	Stratified random sampling	1730	100%		11.6	
33	(42)	2016	Kerman	20.5 (1.5)	Multistage non-random sampling	1730	83.6%		44.6	
34	(38)	2016	Bushehr	22.1 (2.3)	Random sampling	977	100%		16.1	
35	(71)	2014	Karaj	22.4 (4.5)	Random Cluster Sampling	1959	94%	3.4		

a. Survey includes 5 universities from Iran: Tehran University, Isfahan University of Technology, Shahid Bahonar University of Kerman, Razi University of Kermansheh, and Ferdowsi University of Mashhad.

b. Tehran, Guilan, Mazandaran, Golestan, Khorasan shomali, Khorasan razavi, Khorasan Jonobi, Sistan and Balouchestan, Kerman, Hormozgan, Boshehr, Khozestan, Fars, Esfahan, Markazi, Qome, Semnan, Yazad. Qazvin, Lorestan, Chaharmahal and Bakhtiari, Kohgiluyeh Boyer, Kurdistan, Kermansheh, Ilam, Hamedan, Western Azerbaijan, East Azarbaijan, Ardabil, Zanjan university.

c. survey includes 5 universities from whole of Iran: Tehran university, Isfahan University of Technology, Shahid Bahonar University of Kerman, Razi University of Kermansheh, Ferdowsi University of Mashhad

The total sample size in 37 studies that reported the prevalence of water-pipe smoking was 64738 university students. Among the 37 studies, 18 were on the male students (N= 27565) and 16 studies on female student (N=35507), respectively (Table1).

The highest prevalence of water-pipe smoking (at least once in a lifetime) in total male and female students was equal to 41.3% (17), and the lowest prevalence was 6.3% (18) in Shiraz. The highest and the lowest prevalence of water-pipe smoking in male students were 67.2% and 15% respectively (19, 20). In addition, the highest prevalence of water-pipe smoking in female students was reported 42.8% (3) and the lowest prevalence was 7.5% (21).

### Heterogeneity

The result of chi-squared test and I^2^ index indicated that there was a significant between-study heterogeneity in the prevalence of at least once in lifetime water-pipe smoking (*P*<0.001, I^2^ =98.95%), at least once in the last year (*P*<0.001, I^2^=98.84%) and in at least once in the last month (*P*<0.001, I^2^ =99.07%), so we used random effect model in this study.

### Subgroup analysis

According to the random effect model, the pooled prevalence of water-pipe smoking at least once in a lifetime was 25% (95% CI: 22–29) in university student, and the pooled prevalence of water-pipe smoking at least once in a lifetime in male and female was 37% (95%CI: 30–45), 17% (95%CI: 15–19) respectively (Table 2). The pooled prevalence of water-pipe smoking at least once in the last year in mixed gender in college student was 21% (95%CI: 16–25) and in male and female student was 31% (95%CI: 20–42) and 14% (95%CI: 8–20), respectively (Table 2). In addition, the pooled prevalence of water-pipe smoking at least once in the last month in both male and female student was 8% (95%CI: 5–11), and in male and female subgroup was 11% (95%CI: 5–16) and 4% (95%CI: 2–6) respectively (Table 2).

**Table 2:** The pooled prevalence of water-pipe smoking in college students of Iran

***Subgroup***		***No. of Included studies***	***Pooled Prevalence (Random Effect)***	***95% CI***	***I^2^***	**P *value for I^2^***
At least once in the Life time		33	25	22–29	98.95	P<0.001
	Male	12	17	15–19	85.19	P<0.001
	Female	14	37	30–45	99.05	P<0.001
At least once in the Last year		8	21	16–25	98.84	P<0.001
	Male	5	31	20–42	98.60	P<0.001
	Female	5	14	8–20	98.19	P<0.001
At least once in the Last month		12	8	5–11	99.70	P<0.001
	Male	7	11	5–16	98.54	P<0.001
	Female	7	4	2–6	96.90	P<0.001
At least once in the Lifetime by sampling method	Census	2	31	29–33	15.25	P=0.91
	Random Cluster Sampling	5	23	17–30	99.2	P<0.001
	Random Sampling	12	20	14–27	98.8	P<0.001
	Stratified random sampling	8	27	22–33	96.6	P<0.001

Although the pooled prevalence of lifetime water-pipe smoking by sampling method was 31% (95%CI: 29–33) in census sampling method, and in random cluster sampling, random sampling and stratified random sampling method was 23% (95%CI: 17–30), 20% (95%CI: 14–27) and 27% (95%CI: 22–33) respectively (Table 2).

### Sensitivity Analysis

The lower pooled prevalence of water pipe-smoking after omitting the study conducted in Azad Medical University (17) was 24.8% (95%CI: 20.1–28.7), and the higher pooled prevalence estimation in sensitivity analysis was 26.1 (95% CI: 22.4–29.8) after omitted the study in medical students of southern Iran (18). More details of sensitivity analysis in every subgroup are described in Table 3.

**Table 3:** Results of Sensitivity Analysis to Assess the Effects of Every Study on Pooled Prevalence of water-pipe smoking

***Subgroup***	***Pre-Sensitivity Analysis***				***Post-Sensitivity Analysis***		
	No. of Included studies	Upper and Lower of EF^a^	95% CI	Pooled Prevalence (Random Effect)	Excluded Studies	95% CI	Pooled Prevalence (Random Effect)
At least once in the Life time	33	Upper	22–29	25	18	22.47–29.85	26.16
		Lower			17	20.19–28.71	24.81
At least once in the Last year	8	Upper	16–25	21	41	18.46–27.69	23.07
		Lower			42	14.94–20.62	17.78
At least once in the Last month	12	Upper	5–11	8	20	6.12–10.99	8.55
		Lower			43	4.49–10.38	7.43

a. EF: effect size; the upper and lower limit of effect size (pooled odds ratio) in post-sensitivity analysis after omitting each study

### Meta-Regression Analysis

To assess the effect of suspected variables such as year of study, sample size and sampling method in heterogeneity we used meta-regression analysis. Results of meta-regression analysis in Table 4 did not show any significant association between this variable the prevalence of water-pipe smoking.

**Table 4:** Meta-regression analysis for assessing the effect of suspected variables on the pooled Prevalence of Water-pipe smoking

		***Univariable Model***			***Multivariable Model***		
Prevalence rate	Variable	β	SE	*P-*value^*^	β	SE	*P*-value
At least once in the Life time	Sample size^a^	0.27	4.01	0.94	−0.34	4.14	0.93
	Year of study	0.80	4.44	0.85	3.29	5.04	0.52
	Sampling method^b^	−8.10	8.01	0.32	−10.65	9.13	0.25
At least Last year once in the	Sample size	6.34	12.31	0.62	6.8	14.23	0.65
	Year of study	−0.006	9.76	1	1.36	14.23	0.65
	Sampling method	-	-	-	-	-	-
At least once in the Last month	Sample size	4.70	4.43	0.31	9.49	4.73	0.08
	Year of study	−2.65	2.60	0.33	−6.08	3.05	0.08
	Sampling method	−1.07	4.68	0.82	4.19	4.75	.040

a Studies with sample size ≥1,000 versus <1,000 as reference.

b Random sampling, multistage random sampling, stratified random sampling, random cluster sampling vs. census as reference.

* *P*-value<0.05 considered significant

## Discussion

We systematically reviewed the prevalence of hookah smoking among university students in Iran. We found that among 39 studies that reported the prevalence of hookah smoking, the prevalence of lifetime hookah smoking in total, males and females was 24%, 37%, and 17% respectively. Also in the last year, the prevalence of lifetime hookah smoking in total, males, and females was 21%, 31%, and 14% respectively. The results indicated that the prevalence of hookah smoking in the last month was 8%, 11%, and 4 % for total, male, and female students.

To the best of our knowledge, this study is the first try to reporting the pooled prevalence of hookah smoking among Iranian university students. There are a lot of studies that reported prevalence of hookah smoking in other countries especially western ones. In North Carolina lifetime and last month prevalence of hookah smoking among university students has been reported as 40% and 17% respectively (22). Also in the United States, 40.5%, 30.6% and 9.5% of students smoked hookah in the lifetime, last year, and last month (11). In a British university, 38% and 8% of students reported hookah smoking in the lifetime and regularly use (12). The last report of Monitoring the Future (MTF) study indicated that the last year prevalence of hookah smoking decreased from 27.9% in 2011 to 23.4% in 2015 (23). Another study in the United States showed that the prevalence of hookah smoking among university students was 7.8% in the past month (24). The results of the National Collage Health Assessment (NCHA) indicate that among United States university students, the lifetime prevalence of hookah smoking increased from 24.8% to 30.8%, from 2008 to 2010 and last year prevalence ranged from 7% to 10.2% in this time interval (25). The comparison of our findings as a meta-analysis and the prevalence of hookah smoking in western countries indicated that the prevalence of hookah smoking among Iranian university students is slightly lower than in western countries.

In developing countries, the pattern of hookah smoking among university students seems differently from developed ones. For example in South Africa, lifetime and last month prevalence of hookah smoking among university students was 63% and 9.9% respectively in 2013 (26).

The prevalence of hookah smoking in Middle Eastern countries (other than Iran) including Bahrain, Oman, Qatar, United Arab Emirates, Kuwait, Yemen, Lebanon, and Syria ranged from 9% to 15% among the general population (27–29). However, for university students, the rates are different. The result of a review showed that among Eastern Mediterranean Region the lifetime prevalence of hookah smoking among university students was highest in Lebanon 65.3%) (6).

In general, it seems that the prevalence of hook-ah smoking in our study is lower than in other neighboring countries. For example, the lifetime and last month prevalence of hookah smoking have been reported as 61.1% and 42.7% respectively for university students of Jordan (30). In addition, the last month’s prevalence of hookah smoking has been reported as 32.3% among Lebanon university students (31). The total prevalence of hookah smoking was 32.7% among the university of Turkey students (32). The higher rate of hookah smoking in neighboring Arab countries of Iran is due to the cultural differences in these countries that use of hookah was popular in males and females from the ancient period. In addition, there is a misunderstanding in these countries that hookah smoking is healthy, safer, and non-addictive comparing cigarette smoking. The results of a meta-analysis indicated that in comparison to other population, the prevalence of hookah smoking was alarmingly high among university students (33). In Iran, the results of a pooled analysis of National STEPS Surveys indicated that the prevalence of hookah smoking ranged from 1.7% to 10.9% in men and 0 to 16.8% in women (34). This national study also showed that South and Southeastern areas of Iran with similarity in cultural context with neighborhood Arab countries had a high prevalence of daily hookah smoking. As a result, it seems that in consistent with other studies (33), the prevalence rate of hookah smoking among Iranian university students is higher than general population.

There are lots of studies that mentioned the deleterious health outcomes of hookah smoking (35). In addition to these outcomes, hookah smoking is likely to be associated with the risk of dependency (36). Hookah smoking can serve as gateway to engage in cigarette smoking or as a replacement for it among quitters (37). On the other hand, some statistical approaches like latent class analysis showed that hookah smoking was occurring commonly with cigarette smoking among some specific groups of university students (38, 39). From preventive view, it is better to consider integrated programs to reducing tobacco smoking among Iranian university students.

The results of a review showed that socializing, relaxation, pleasure, and entertainment are the most important motives for hookah smoking among the general population. On the other hand, additional motives were reported by university students and include peer pressure, fashion, and curiosity (35). Health policymakers should scale up efforts to combat the hookah smoking phenomenon in Iran. The luck of media campaigns about hookah smoking implied that they must be safer (40). Also, there is a need to develop a valid and reliable questionnaire for assessing Iranian university student’s motives, beliefs and attitudes toward hookah smoking. Such a tool may be very useful to identify factors that could modify the effect of future interventions designed for hookah smoking cessation.

Our study is unique in full consideration of all types of frequency and duration of waterpipe use in a college student in Iran e.g. lifetime use, last year use, last month and daily use. Moreover, we tried to consider the gray literature to cover all studies conducted in Iran. However, this study has several limitations regarding the high level of heterogeneity existed in the subgroups pooled prevalence and should be considered when interpreted. Some variations in studies could be the source of heterogeneity e.g. sampling strategy and representativeness of samples, the sample size of studies, the validity of questionnaires used in studies. We tried to include all gray literature in the meta-analysis; however, full consideration of gray literature is another limitation in this study due to the lack of universal databases in Iran to access all research projects and dissertations.

## Conclusion

The prevalence of hookah smoking in our study is lower than in other neighboring countries. Gender and methods of sampling are associated with variation in the pooled prevalence of hook-ah smoking in our study. The prevalence rate of hookah smoking among Iranian university students is higher than the general population. Regarding lower stigmatization of hookah than cigarette smoking in Iranian culture and increasing the prevalence of hookah in college students than general population, some national preventing programs such as peer group educations program in universities, mass media campaigns and comprehensive nationwide tobacco control programs is needed.

## Ethical considerations

Ethical issues (Including plagiarism, informed consent, misconduct, data fabrication and/or falsification, double publication and/or submission, redundancy, etc.) have been completely observed by the authors.
